# Trends in the U.S. Direct Care Workforce, 2013 to 2023

**DOI:** 10.1093/geroni/igaf122.2138

**Published:** 2025-12-31

**Authors:** Christopher Kelly, Jerome Deichert

**Affiliations:** University of Nebraska Omaha, Omaha, Nebraska, United States; University of Nebraska Omaha, Omaha, Nebraska, United States

## Abstract

We used data from the 2013-2023 American Community Survey to examine trends among direct care workers (DCWs) in both occupation and industry. We found the number of medical aides (i.e., nursing assistants, psychiatric aides, and home health aides) decreased 20.9%, from 2.31 million in 2013, to 1.83 million in 2023, while the number of nonmedical aides (i.e., personal and home care aides) increased 19.2%, from 1.19 million to 1.42 million over the same 10-year period. Medical aides were more likely than nonmedical aides to work in an institutional setting (i.e., hospitals, nursing homes, assisted living), to be under age 25, to be Latina(o), to be African American, and to be never married or recently married. From 2013 to 2023, there were increases in the number of DCWs employed by home health care services (8.9%) and individual and family services (34.4%). There were decreases in the number of DCWs employed by skilled nursing facilities (35.1%), outpatient care centers (29.0%), hospitals (20.0%), assisted living facilities (10.1%), and private households (15.6%). DCWs employed in institutional settings were more likely to be medical aides, to be male, to be under age 25, to be African American, to be never married, and to work year-round and full-time. These findings suggest two major shifts in the direct care workforce between 2013 and 2023. Direct care workforce jobs are trending towards non-medical aides and to home and community-based services. Further, the DCWs employed as medical or non-medical aides, or in institutional or non-institutional settings, are increasingly distinct.

